# Serum Calcium Levels and Parkinson’s Disease: A Mendelian Randomization Study

**DOI:** 10.3389/fgene.2020.00824

**Published:** 2020-08-11

**Authors:** Yanchao Wang, Luyan Gao, Wenjing Lang, He Li, Pan Cui, Nan Zhang, Wei Jiang

**Affiliations:** ^1^Department of Neurology, Tianjin Neurological Institute, Tianjin Medical University General Hospital, Tianjin, China; ^2^Department of Neurology, Affiliated Hospital of Chifeng University, Chifeng, China; ^3^Department of Neurology, The Fourth Central Clinical College of Tianjin Medical University, Tianjin, China

**Keywords:** Parkinson’s disease, serum calcium, Mendelian randomization, pleiotropy analysis, power analysis

## Abstract

**Background:**

Though increasing epidemiological studies have evaluated the correlation between serum calcium contents and Parkinson’s disease (PD), the results are inconsistent. At present, whether there is a causal association between serum calcium content and PD remains undetermined.

**Objective and Methods:**

This study was designed to explore the relationship between increased serum calcium contents and PD risk. In this present study, a Mendelian randomization trial was carried out using a large-scale serum calcium genome-wide association study (GWAS) dataset (*N* = 61,079, Europeans) and a large-scale PD GWAS dataset (*N* = 8,477, Europeans including 4,238 PD patients and 4,239 controls). Here, a total of four Mendelian randomization methods comprising weighted median, inverse-variance weighted meta-analysis (IVW), MR-Egger, and MR-PRESSO were used.

**Results:**

Our data concluded that genetically higher serum calcium contents were not significantly related to PD.

**Conclusion:**

In conclusion, we provided genetic evidence that there was no direct causal relationship between serum calcium contents and PD. Hence, calcium supplementation may not result in reduced PD risk.

## Introduction

Previous studies indicated that calcium is involved in many biological processes, and altered calcium homeostasis is widely regarded as a basis for cognitive deficits in normal subjects and certain neurodegenerative diseases (LaFerla, 2002; Larsson et al., 2017). Parkinson’s disease (PD) is the second most common neurodegenerative disorder, which affects 1–2% people older than 65 years (Gibrat et al., 2009; Saad et al., 2011; Liu et al., 2015a, b; Liu G. et al., 2016). Particularly, emerging epidemiological research has evaluated the correlation between serum calcium contents and PD, and calcium dysregulation has been found in PD (Abou-Raya et al., 2009; Meamar et al., 2013; Schapira, 2013; Liu J. et al., 2016). However, the results reported by these studies are often inconsistent.

In 2009, Abou-Raya et al. (2009) evaluated the relationship between bone changes and PD. In their study, the bone density and mineral metabolism in 82 PD patients and 68 age- and sex-matched controls were measured, of which the results showed that serum calcium and vitamin D levels were significantly decreased in PD patients compared with controls. Similarly, Meamar et al. (2013) compared serum calcium contents from 105 PD patients and 112 matched controls in 2013, and indicated that serum calcium contents were significantly decreased in PD patients relative to controls. In addition, Liu J. et al. (2016) also found that serum calcium contents were significantly decreased in 77 PD patients with dementia compared to 75 healthy control subjects in 2016. It should be noted that no significant difference in serum calcium content was observed between the PD group without dementia and the healthy control group in their study.

At present, it remains unclear whether there is a causal relationship between serum calcium content and PD. Here, we tried to find the causality between the serum calcium levels and PD, thereby providing effective therapies for PD. Recently, Mendelian randomization methods have been widely applied to assess causal relationships through genome-wide association study (GWAS) datasets (Mokry et al., 2015; Nelson et al., 2015; Larsson et al., 2017; Manousaki et al., 2017; Cheng et al., 2018, 2019; Hu et al., 2018; Liu et al., 2018, 2019; Sun et al., 2019; Zhuang et al., 2019a, b). Therefore, a Mendelian randomization study was carried out to explore the genetic relationship between serum calcium content and PD through the large-scale serum calcium and PD GWAS datasets in our study.

## Materials and Methods

### Study Design

A previous study reported that human genetic variants are randomly allocated, which is the basis of the Mendelian randomization method (Emdin et al., 2017). These genetic variants are mostly independent from confounding variables and can be regarded as instrumental proxies to assess the causal relationship between serum calcium levels and its outcome. Mendelian randomization was conducted according to three primary assumptions as described in the previous reports (Emdin et al., 2017; Larsson et al., 2017). Assumption (1): the selected genetic variants are related to serum calcium levels; assumption (2): these genetic variants are not related to confounders; assumption (3): these genetic variants affect PD risk only via serum calcium levels. Meanwhile, assumptions (2 and 3) were together regarded as independence from pleiotropic effects. Our present work was performed using the publicly available large-scale GWAS dataset. Informed consent from all the participants was obtained in all the original research.

### Serum Calcium GWAS Dataset

In this study, eight genetic variants influencing serum calcium content with genome-wide significance (*P* < 5.00E−08) were used as the instrumental proxies (O’Seaghdha et al., 2013). The dataset contained 39,400 subjects deriving from 17 population-based cohorts in the discovery stage and 21,679 subjects in the replication stage (*N* = 61,079, Europeans). All the genetic variants were distributed at various genes and were not in linkage disequilibrium as presented in Table 1. The measurement of serum calcium content was described in details in Additional File 1.

**TABLE 1:** Characteristics of 8 genetic variants in serum calcium and PD GWAS datasets.

SNP	Chr	Nearby genes	EA^a^	NEA	EAF^b^	Serum calcium GWAS			Discovery PD GWAS			Validation PD GWAS		
						Beta (mg/dL)^c^	SE^c^	*P-*value^c^	Beta^d^	SE^d^	*P*-value^d^	Beta^d^	SE^d^	*P*-value^d^
rs780094	2	GCKR	T	C	0.42	0.017	0.003	1.30E−10	–0.0037	0.0332	0.911	–0.0190	0.0233	0.4156
rs1550532	2	DGKD	C	G	0.31	0.018	0.003	8.20E−11	–0.0411	0.0354	0.2458	–0.0547	0.0246	0.0265
rs1801725	3	CASR	T	G	0.15	0.071	0.004	8.90E−86	0.0425	0.046	0.3547	0.0421	0.0334	0.2052
rs10491003	10	GATA3	T	C	0.09	0.027	0.005	4.80E−09	–0.0427	0.0566	0.4501	–0.0564	0.0399	0.1576
rs7336933	13	DGKH/KIAA0564	G	A	0.85	0.022	0.004	9.10E−10	–0.0352	0.046	0.4441	0.0564	0.0321	0.0788
rs1570669	20	CYP24A1	G	A	0.34	0.018	0.003	9.10E−12	–0.0303	0.0342	0.3757	–0.0051	0.0240	0.8318
rs7481584	11	CARS	G	A	0.7	0.018	0.003	1.20E−10	0.0429	0.0365	0.24	0.0018	0.0251	0.9430
rs17711722	7	VKORC1L1	T	C	0.47	0.021	0.003	2.80E−11	0.0714	0.0331	0.03075	0.0266	0.0228	0.2483

*PD, Parkinson’s disease; GWAS, genome-wide association studies; SNP, single-nucleotide polymorphism; Chr, chromosome; EA, Effect Allele; NEA, Non-Effect Allele; EAF, Effect Allele Frequency; SE, standard error. ^a^Effect allele (Serum calcium raising allele). ^b^Frequency of the effect allele (serum calcium raising allele) in the GWAS dataset of serum calcium (O’Seaghdha et al., 2013). ^c^Beta (mg/dL) is mainly based on the effect allele (serum calcium raising allele) as a regression coefficient. Beta < 0 represents that the effector allele regulates the decrease in serum calcium levels and Beta > 0 represents that the effector allele regulates the increase in serum calcium levels. The summary statistics of Beta, SE and P-value are obtained from the serum calcium GWAS dataset (O’Seaghdha et al., 2013). ^d^Beta is mainly based on the effect allele (serum calcium raising allele) as a regression coefficient. Beta < 0 represents that the effector allele regulates a decrease in PD risk and Beta > 0 represents that the effector allele regulates an increase in PD risk. Beta = ln(odd ratio), and represents the overall estimated effect size for the serum calcium raising allele. We obtained summary statistics of Beta, SE and P-value from the discovery and validation PD GWAS datasets of European descent (Pankratz et al., 2012; Liu et al., 2017).*

### PD GWAS Dataset

Discovery PD GWAS dataset was derived from a meta-analysis of five independent PD GWAS datasets (n = 8,477, Europeans including 4,238 PD patients and 4,239 controls) (Pankratz et al., 2012). The relationship between each dataset and PD susceptibility was tested using a logistic regression model, and the results of each dataset were performed for meta-analysis. Here, we used the meta-analysis results of about 2,525,704 SNPs. Validation of the PD GWAS dataset was derived from a case control-associated map obtained from family history of disease in the UK Biobank (Liu et al., 2017). In brief, this GWAS dataset consisted of 4,627 PD cases and 109,826 controls, all of which are of European ancestry. Here, we used the genome-wide analyzed results by proxy. Informed consent from all the participants was obtained in all the original research. Link for the serum calcium and PD GWAS datasets is https://www.ebi.ac.uk/gwas/.

### Pleiotropy Analysis

For Mendelian randomization studies, a crucial problem is potential contradiction of assumptions (2, 3) via pleiotropic effects which occur when a genetic instrument is related to the outcome, independent of the exposure. In this work, a pleiotropy evaluation was conducted to confirm that the eight genetic variants did not affect PD risk via biological pathways outside serum calcium content, and three steps were performed to mitigate the risk of pleiotropy.

In 2016, a major review from the Lancet Neurology reported that PD risk factors included body mass index (BMI), diabetes, blood cholesterol, hypertension, alcohol, vitamins, fat and other micronutrients, and PD protective factors included smoking, coffee, tea and serum urate (Ascherio and Schwarzschild, 2016). At stage 1, we evaluated the potential pleiotropy using some known confounders including major lipids (total cholesterol and triglyceride, low density lipoprotein, high density lipoprotein, etc.), type 2 diabetes, hypertension, blood pressure, BMI, waist circumference, hip circumference and waist-to-hip ratio, smoking, alcohol consumption, serum urate and vitamin D levels. The significance threshold for the relationship between the eight variants and the above-mentioned confounders was defined to be a Bonferroni-corrected P < 0.05/8 = 0.00625.

Previous studies indicated that a statistical method, termed MR-Egger, could provide a reliable evaluation for the instrumental variable assumption, and check for the presence of potential pleiotropy (Dale et al., 2017; Tillmann et al., 2017). Therefore, at stage 2, the MR-Egger was used to assess potential pleiotropic relationships between the genetic variants and confounders.

At stage 3, a recently developed statistical method, Mendelian randomization pleiotropy residual sum and outlier (MR-PRESSO) (Verbanck et al., 2018), was used to confirm the horizontal pleiotropic outliers.

### Mendelian Randomization Analysis

In this study, Mendelian randomization analysis was conducted through four widely used methods, including MR-Egger, MR-PRESSO, inverse-variance weighted meta-analysis (IVW) and weighted median (Burgess et al., 2017; Verbanck et al., 2018), which are based on different assumptions and are useful for examining the robustness with each other. The details for the MR-Egger, IVW and weighted-median methods have been described in our previous study (Liu et al., 2018).

The IVW method assumed that all genetic variants conformed to the instrumental variable assumption, and the MR-Egger method assumed that these instrumental variables were not constant. A weaker hypothesis, the InSIDE assumption (Instrument Strength Independent of Direct Effect assumption), could still provide a consistent causal effect estimate (Bowden et al., 2015). In addition, the MR-PRESSO method was proven to require a basic assumption that more than half of the genetic variants were qualified instrumental variables and satisfied both the balanced pleiotropy and InSIDE assumption (Verbanck et al., 2018). Finally, the weighted-median method can estimate a consistent causal effect when more than half of the genetic variants are effective instrumental proxies (Bowden et al., 2016). The odds ratio (OR) and 95% CI of PD corresponded to per 0.5 mg/dL elevation [1 standard deviation (SD)] in serum calcium content. All analyses were performed through the R packages “MR-PRESSO” (Verbanck et al., 2018) or “Mendelian Randomization” (Yavorska and Burgess, 2017).

For the statistical analysis of the genetic association of serum calcium content with PD risk, P < 0.05 was considered as significant. In addition, a sensitivity analysis was conducted to evaluate the robustness of the causal estimates. Subsequently, each genetic variant was excluded one by one from the MR analysis via the leave-one-out permutation analysis, to assess the impact of each genetic variant on the causal estimates.

### Power Analysis

Serum calcium variance (R^2^) proportion was assessed with the following formula:

$$R^{2}=\sum_{i=1}^{K}\frac{\beta_{i}^{2}*2*M\,A\,F_{S\,N\,P_{i}}\,\left(1-M\,A\,F_{S\,N\,P_{i}}\right)}{v\,a\,r\,\left(X\right)}$$

Where β_*i*_ is the effect size (beta coefficient) associated with the serum calcium content for *SNP*_*i*_, *MAF*_*SNP_i*_is the minor allele frequency for *SNP*_*i*_, *K* represents genetic variant number, and*var*(*X*) represents serum calcium variance (*var*(*X*) = *SD*^2^, and 1 *SD* = 0.5 mg/dL).

The instrumental variable strength (genetic variant related to serum calcium content) was assessed through the first-stage F-statistics. Generally, a threshold (*F* > 10) was used in case of bias in Mendelian randomization trials (Burgess and Thompson, 2011). Here, we calculated the F-statistics and statistical power using a web-based tool mRnd^1^ and a two-sided type-I error rate α of 0.05 (Brion et al., 2013).

## Results

### Association Between Serum Calcium Variants and PD

From the discovery and validation PD GWAS datasets, eight genetic variants associated with serum calcium levels were extracted for statistical analysis. As shown in Table 1, the results suggested that all these genetic variants were not significantly related to PD (*P* > 0.05/8 = 0.00625).

### Pleiotropy Analysis

At stage 1, the results showed that rs780094 and rs1570669 variants were significantly related to some known confounders (*P* < 0.05/8 = 0.00625). More specifically, rs780094 was significantly related to low density lipoprotein (*P* = 1.02E−07), high density lipoprotein (*P* = 2.67E−03), total cholesterol (*P* = 5.28E−41), triglyceride (*P* = 7.08E−125), type II diabetes (*P* = 1.00E−05), alcohol consumption (*P* = 3.65E−09), hip circumference (*P* = 3.40E−05) serum urate (*P* = 6.52E−39) and waist-to-hip ratio adjusted for BMI (*P* = 1.80E−03); rs1570669 was significantly related to circulating vitamin D (*P* = 5.33E−06). Hence, we excluded rs780094 and rs1570669 variants to meet the assumptions of the Mendelian randomization design. More detailed datasets were provided in Additional File 2. At stage 2, using the remaining six genetic variants (excluding rs780094 and rs1570669 variants identified at stage 1), the MR-Egger intercept test demonstrated that no evidence of pleiotropy was detected in the PD discovery GWAS dataset, with intercept = 0.000 and *P* = 1.00, or in the PD validation GWAS dataset, intercept = −0.021 and *P* = 0.562. At stage 3, no horizontal pleiotropic outliers were identified in either the discovery or validation PD GWAS datasets by MR-PRESSO. Hence, our Mendelian randomization trial focused on the remaining six genetic variants.

### Association Between Serum Calcium Content and PD Risk

For the PD discovery dataset, the IVW analysis showed that per SD increase in serum calcium content (0.5 mg/dL) was not causally associated with a decreased PD incidence (OR = 1.73, 95% CI: 0.47–6.37, *P* = 0.408) using the remaining 6 genetic variants. Likewise, the weighted-median estimate (OR = 1.72, 95% CI: 0.52–5.66, *P* = 0.374), MR-Egger estimate (OR = 1.73, 95% CI: 0.12–25.9, *P* = 0.691) and MR-PRESSO estimate (OR = 1.73, 95% CI: 0.47–6.37, *P* = 0.446) did not show a significant causal association of serum calcium content with PD risk (*P* < 0.05 was considered as significance). For the PD validation dataset, our study demonstrated a similar conclusion that neither the IVW estimate (OR = 1.39, 95% CI: 0.45–4.31, *P* = 0.564), weighted-median estimate (OR = 1.78, 95% CI: 0.76–4.15, *P* = 0.185), MR-Egger estimate (OR = 2.46, 95% CI: 0.25–23.9, *P* = 0.437) or MR-PRESSO estimate (OR = 1.39, 95% CI: 0.45–4.30, *P* = 0.589) demonstrated that the increased serum calcium content was significantly related to PD risk. The details are shown in Table 2. Furthermore, the leave-one-out permutation analysis indicated the direction and precision of causal association between enhanced serum calcium content and increased PD risk was largely unchanged among these methods. In addition, we provide the individual estimates from different methods inferring the causality between each of serum calcium genetic variants and PD in the PD discovery and validation datasets, respectively (Additional Files 3, 4). The forest plot for MR estimates about the causal effect of genetically increased serum calcium levels on PD using IVW method is also added (Additional Files 5, 6).

**TABLE 2:** Mendelian randomization analysis using four methods.

Dataset	Method	OR	SE	95% CI	*P-*value
PD discovery dataset	Weighted_median	1.72	0.61	0.52–5.66	0.374
PD discovery dataset	IVW	1.73	0.66	0.47–6.37	0.408
PD discovery dataset	MR-Egger	1.73	1.38	0.12–25.9	0.691
PD discovery dataset	MR-PRESSO	1.73	0.66	0.47–6.37	0.446
PD validation dataset	Weighted_median	1.78	0.43	0.76–4.15	0.185
PD validation dataset	IVW	1.39	0.58	0.45–4.31	0.564
PD validation dataset	MR-Egger	2.46	1.16	0.25–23.9	0.437
PD validation dataset	MR-PRESSO	1.39	0.58	0.45–4.30	0.589

*SE, standard error; OR > 0 and OR < 0 means that high serum calcium levels increase and reduce the risk of PD. OR, odds ratio; CI, confidence interval; IVW, Inverse-variance weighted meta-analysis. The association between serum calcium levels and PD was at the significance level P < 0.05.*

### Power Analysis

Here, all these six genetic variants could explain about 0.81% of the serum calcium variances (*R*^2^ = 0.81%). In both the PD discovery and validation GWAS datasets, the first-stage F-statistics for the instrument included six genetic variants with F more than 10, so there was no instrument bias. Our Mendelian randomization trial had 80% power to determine the impact size of moderate magnitudes with ORs > = 1.95 and 1.48 per SD (0.5 mg/dL) increases in serum calcium content for PD risk in the PD discovery and validation GWAS dataset, respectively.

## Discussion

Calcium (Ca^2+^) is a crucial second messenger that participates in a variety of cellular physiological processes. Particularly in neuronal cells, intracellular Ca^2+^ signaling is under extremely precise control to ensure the smooth functioning of many electrophysiological activities such as neuronal excitability, neurotransmitter secretion and synaptic plasticity. As a result, dysregulation of Ca^2+^ signaling is implicated in neurodegeneration disorders such as Alzheimer’s disease, PD and Huntington’s disease. Recently, the causality between genetically higher serum calcium and reduced AD incidence has been established (Jiang et al., 2018).

It has been well recognized that Ca^2+^ signaling abnormalities play a crucial role in PD pathogenesis. The coordinated regulation of Ca^2+^ fluxes is compromised in PD, which induces selective toxicity in the dopaminergic neurons in the substantia nigra. PD risk is significantly reduced in hypertensive patients treated with L-type Ca^2+^ channel antagonists (Becker et al., 2008; Ritz et al., 2010; Pasternak et al., 2012). The voltage gated L-type Ca^2+^ channel inhibitor isradipine that blocks Ca^2+^ entry can protect SNc neurons in mouse models of PD (Ilijic et al., 2011). A phase III clinical trial of isradipine is in progress to evaluate if it can also benefit PD patients (Liss and Striessnig, 2019). However, the relationship between serum calcium and PD has not been well recognized by observational studies. Here, we investigated the genetic associations of serum calcium content with PD risk using the Mendelian randomization method, and the results were negative. Our results are comparable to the previous epidemiological reports, demonstrating that calcium supplementation was not associated with PD risk (Chen et al., 2007; Mischley et al., 2017). The relationship between altered serum calcium content and PD reported by a few observational studies might be mediated by some confounding factors such as daily physical activity. In conclusion, our results did not support the supplementation of calcium for preventing PD in the general population. Future clinical trials regarding this topic may fail to produce positive benefits.

Mendelian randomization research has many advantages. Firstly, the study can use the large-scale serum calcium and PD GWAS datasets to provide enough power, thereby detecting genetic associations of serum calcium content with PD. Secondly, these serum calcium and PD GWAS datasets were all of European descent, reducing population stratification effects. Thirdly, various independent genetic variants were used as instrument variants to decrease linkage disequilibrium effects. Fourthly, multiple methods were carried out for a comprehensive pleiotropy analysis to rule out two genetic variants related to potential confounding factors. Causal inference results should not depend on a single method. A previous study indicated that if the results were confirmed using a variety of methods, the causal findings might be more reliable, especially if these methods are based on different assumptions (Burgess et al., 2017). Therefore, we chose 4 different methods to test causal effects in this present study. The application of different methods under different situations may affect the results of the causal effect analysis. Specifically, when investigating whether there is a dose-response relation between the genetic instruments and their outcomes, the MR-Egger method is more effective than the IVW or weighted-median methods. However, when exploring the possible effects of outlying genetic variants on causality, the IVW estimates may be less severely affected by other factors compared to the MR-Egger estimate (Burgess and Thompson, 2017). In practical applications, it is recommended to use a series of sensitivity analysis methods. All these mentioned advantages could protect against violations of Mendelian randomization assumptions.

Meanwhile, this Mendelian randomization trial also has some limitations. Firstly, the scale of PD GWAS dataset used by us was small, with 8,477 individuals (Pankratz et al., 2012; Liu et al., 2017). In 2014, Nalls et al. (2014) conducted a large-scale GWAS meta-analysis containing 13708 PD cases and 95,282 controls from 15 independent GWAS datasets in European. However, this dataset was not publicly available, so our trial needs to be repeated when this dataset is available. Secondly, population stratification was not absolutely excluded due to its impacts on the assessment. Thirdly, the genetic relationship between serum calcium content and PD might be different due to the various ethnicities and ancestries, so the genetic relationship needs to be further assessed in other ethnicities and ancestries. However, other meta-analyses, such as meta-analysis based on the Bayesian method (Moreno et al., 2014), have also been used to test this kind of relationship. We will further conduct this meta-analysis in the next study to explore the correlation between serum calcium levels and PD and compared it with our Mendelian randomized analysis. Ultimately, there were some difficulties calculating the false positive rates of different methods and we indeed need to address these concerns. We will examine all the concerns mentioned above in future research.

In conclusion, our data indicated that elevated serum calcium levels were not causally related to PD risk. Hence, calcium supplementation might not contribute to reducing PD risk.

## Data Availability Statement

Publicly available datasets were analyzed in this study. This data can be found here: https://www.ebi.ac.uk/gwas/.

## Author Contributions

YW and LG designed the study, analyzed the data, and wrote the first draft of the manuscript. PC revised the manuscript. WL, HL, and PC collected the data and provided technological support. WJ conceived the study, formulated the research concept, and revised the manuscript. NZ provided suggestions for the study design, revision and proof of the manuscript. All authors contributed to the interpretation of the results and critical revision of the manuscript for important intellectual content and approved the final version of the manuscript.

## Conflict of Interest

The authors declare that the research was conducted in the absence of any commercial or financial relationships that could be construed as a potential conflict of interest.

## Footnotes

1 https://cnsgenomics.shinyapps.io/mRnd/

## Supplementary Material

FILE S1 Methods to measure serum calcium levels in the original study.

FILE S2 *P-*values for associations of 8 calcium-associated genetic variants with known PD risk factors.

FILE S3 Individual causal estimates from each of serum calcium genetic variants and PD using different methods in Discovery PD GWAS dataset.

FILE S4 Individual causal estimates from each of serum calcium genetic variants and PD using different methods in Validation PD GWAS dataset.

FILE S5 Forest plot for MR estimates about the association between genetically increased serum calcium levels and PD in Discovery PD GWAS dataset.

FILE S6 Forest plot for MR estimates about the association between genetically increased serum calcium levels and PD in Validation PD GWAS dataset.

## Abbreviations

BMI: Body Mass Index
CI: confidence interval
GWAS: genome-wide association study
HDL: high density lipoprotein
IVW: inverse-variance weighted
MAF: Minimum allele frequency
MR: Mendelian randomization
MR-PRESSO: Mendelian randomization pleiotropy residual sum and outlier
OR: odds ratio
PD: Parkinson’s disease
SD: standard deviation
SNP: single-nucleotide polymorphism.
