# Supplementary material for: Serum Calcium Levels and Parkinson’s Disease: A Mendelian Randomization Study
Source: Front Genet. 2020 Aug 11;11:824. doi: 10.3389/fgene.2020.00824 (PMC7431982; doi:10.3389/fgene.2020.00824)
Supplement: FILE S1 — Methods to measure serum calcium levels in the original study. [file Table_1.doc]

**Additional file 1**. Methods to measure serum calcium levels in the original study

**Additional file 2**. *P* Values for Associations of 8 Calcium-Associated Genetic Variants with known PD risk factors

**Additional file 3**. Individual causal estimates from each of serum calcium genetic variants and PD using different methods in Discovery PD GWAS dataset.

**Additional file 4**. Individual causal estimates from each of serum calcium genetic variants and PD using different methods in Validation PD GWAS dataset.

**Additional file 5**. Forest plot for MR estimates about the association between genetically increased serum calcium levels and PD in Discovery PD GWAS dataset.

**Additional file 6**. Forest plot for MR estimates about the association between genetically increased serum calcium levels and PD in Validation PD GWAS dataset.

**Additional file 1**. Methods to measure serum calcium levels in the original study ^1^

| Study Name | Study Design | Genotyped sample size | Study exclusions or disease enrichment | Exclusions | Calcium Measurement + QC |
| --- | --- | --- | --- | --- | --- |
| **Discovery cohorts** |  |  |  |  |  |
| Age Gene/Environment Susceptibility Reykjavik Study (AGES) | Population based | 3664 | none | Sample exclusion criteria included sample failure, genotype mismatch with reference panel, and sex mismatch, resulting in clean genotype data on 3,219 individuals. | Serum calcium was measured using a colorimetric assay on a Hitachi 912 using a Roche Diagnostics assay . |
| ARIC | Prospective, population-based(1) | 9713 of European ancestry | none | Of the 9713 genotyped individuals of European ancestry, we excluded 658 individuals based on discrepancies with previous genotypes, disagreement between reported and genotypic sex, one randomly selected member of a pair of first-degree relatives, or outlier based on measures of average DST or more than 8 SD away on any of the first 10 principal components. | Total serum calcium was measured at ARIC visit 1 (1987-89) using a colorimetric method on a DACOS analyzer (http://www.cscc.unc.edu/aric/visit/Clinical_Chemistry_Determinations.1_10.pdf). |
| BLSA | Prospective population based study | 1230 | none | Non-European ancestry based on Eigenstrat (N=368), call rate <98.5 (N=5), sex misspecification (N=9), Missing calcium data (N=129) | Serum calcium was measured using a colorimetric assay. |
| Cohorte Lausannoise (CoLaus) | Population-based cross-sectional study | 5435 | none | Individuals with genotyping efficiency <90% were removed. Some "measured" SNPs with missing values for some individuals were imputed. As a result, the rSqHat < 1. | For each CoLaus participant a venous blood sample was collected under fasting conditions. Total serum calcium was measured by O-cresolphtalein (2.1% –1.5% maximum inter and intra-batch CVs); albumin was measured by bromocresol green (2.5% – 0.4%). |
| CROATIA-Vis | Family-based, cross-sectional study in an isolated population | 924 | none | Exclusions: sample call rate<95% | Serum calcium was measured using a colorimetric assay. |
| CROATIA-Korcula | Family-based, cross-sectional study in an isolated population | 899 | none | Exclusions: sample call rate<97% | Serum calcium was measured using a colorimetric assay. |
| CROATIA-Split | Population-based, cross-sectional study | 499 | none | Exclusions: sample call rate<97% | Serum calcium was measured using a colorimetric assay. |
| Framingham Heart Study (FHS) | Prospective family-based | 9300 | none | Of the 9,274 participants who underwent genotyping, we made the following exclusions: sample call rate <97% (n=666), genotype heterozygosity > 5 standard deviations, and ambiguous family data (n=127). This resulted in a total of 8,481 genotyped individuals. | Serum calcium was measured using a colorimetric assay. |
| HABC | prospective cohort study | 1663 | none | none | Serum calcium was measured using a colorimetric assay. |
| InCHIANTI | Prospective population based study | 1231 | none | Ambiguous family data (N=4), call rate < 98.5% (N=12), sex misspecification (N=1), heterozygosity>0.3 (N=4), Missing calcium data (N=6) | Serum calcium was measured using a colorimetric assay. |
| Lothian Birth Cohort 1936 (LBC1936) | Population based birth cohort | 1005 | none | Individuals with a disagreement between genetic and reported gender were removed (n = 12). Relatedness between subjects was investigated and, for any related pair of individuals, one was removed [PI_HAT (proportion of IBD) > 0.25, n = 8). Samples with a call rate ≤ 0.95 (n = 16), and those showing evidence of non-European descent by multidimensional scaling, were also removed (n = 1). | Serum calcium was measured using a colorimetric assay. |
| London Life Sciences Population (LOLIPOP) study | | |  |  |  |
| LOLIPOP EW A | Population based prospective cohort study | 878 | none | excluded samples of duplicates, contaminated samples, call rate <%95, and the same samples apeared in EW610 | Serum calcium was measured using standard approach |
| LOLIPOP EW P | Population based prospective cohort study | 1005 | none | excluded samples of duplicates, contaminated samples, call rate <%95, and the same samples apeared in EW610 | Serum calcium was measured using standard approach |
| LOLIPOP EW610 | Population based prospective cohort study | 945 | none | excluded samples of duplicates, call rate <%95, relatedness, gender discrepancy, ethnic outliers, and imcomplete clinical data | Serum calcium was measured using standard approach |
| Ogliastra Genetic Park - Talana Study | Population-based study with pedigree information | 860 | none |  | Calcium levels were determined with an automated Targa BT-3000 Chemistry Analyser ( ml/dL) |
| ORCADES | Family-based, cross-sectional study in an isolated population | 889 | none | Exclusions: sample call rate<97% | Serum calcium was measured using a colorimetric assay. |
| SHIP | Prospective population-based study | 4081 | none | Excluded arrays with CallRate < 92%, duplicate samples (by estimated IBD) and individuals with reported/genotyped gender mismatch | Non-fasting blood samples were drawn from the cubital vein in the supine position between 7.00 a.m. and 7.00 p.m. The measurement of total calcium concentration was performed immediately after blood withdrawal. Samples were analysed on the Hitachi 911 by o-cresolphthalein complexone colorimetry (Boehringer Mannheim, Germany). The internal quality controls were analyzed daily. During the course of the study the inter as well as the intra-assay coefficient of variation was <5%. In addition, the laboratory takes part quarterly in the official national German external proficiency testing programs and fulfilled the requirements. |
| The Rotterdam Study (RS) | Prospective population based study | 5974 | NA | Any samples with a call rate below 97.5%, excess autosomal heterozygosity >0.336 (~FDR <0.1%), mismatch between called and phenotypic gender, or if there were outliers identified by the IBS clustering analysis (see below) with >3 standard deviations from population mean or IBS probabilities >97% were excluded from the analysis | Serum calcium was measured at baseline visit in the Rotterdam Study with a colorimetric detection assay using the Hitachi 917 (Roche, Mannheim, Germany). |
| The Cardiovascular Health Study (CHS) | Prospective, population-based | 3,329 CHS Caucasian participants | A total of 1908 persons were excluded from the GWAS study sample due to the presence at study baseline of coronary heart disease, congestive heart failure, peripheral vascular disease, valvular heart disease, stroke or transient ischemic attack or lack of available DNA. | The present report is based upon genotyping results from 3,329 CHS Caucasian participants, who were free of clinical cardiovascular disease at baseline, consented to genetic testing, and had DNA available for genotyping. Genotypes were called using the Illumina BeadStudio software. Genotyping was successful in 3,291 persons. | Serum calcium has been measured in unit mg/dL |
| **Replication cohorts** |  |  |  |  |  |
| Bus Santé study | Cross-sectional population-based study | 5622 | none | Of the 5,622 participants who underwent genotyping, genotyping was unsuccessfull for 2.7% (N=151). This resulted in a total of 5,471 genotyped individuals. Analyses were restriced to Caucasians. Caucasian was defined as self-reported citizenship corresponding to South/North America, Europe, and Australia regions | Serum calcium was measured using a colorimetric assay using Arsenazo-III reactive (Architect CI4100®, Abbott). Coefficient of variation (CV) = 5.4% |
| INGI-Carlantino-Project | Isolated population | 679 | none | we made the following exclusions: sample call rate <97% | Serum calcium was measured using a colorimetric assay. |
| INGI-FVG-Project | Isolated population | 1471 | none | we made the following exclusions: sample call rate <97% | Serum calcium was measured using a colorimetric assay. |
| INGI-CILENTO | Cross-sectional population based | 1147 | none | none | Serum calcium was measured using a colorimetric assay. |
| KORA F3 Study (Cooperative Health Research in the Region of Augsburg) | Population-based | 1643 | none | 3, because of no available information on serum calcium. This resulted in a total of 1640 individuals | Serum calcium was measured using a colorimetric assay. |
| KORA F4 Study | Population-based | 1814 | none | 5, because of no available  information on serum calcium. This resulted in a total of 1809 individuals | Serum calcium was measured using a colorimetric assay. |
| LURIC Study | Case-control | 3032 |  | sample call rate <95%, gender ambiguity, relatedness. This resulted in a total of 2927 genotyped individuals. | o-Kresolphthalein-complexon, CA/Hitachi 717, Roche, Germany |
| PIVUS | Prospective cohort | 958 | none | Sample call rate <95%, genotype heterozygosity > +-3 standard deviations, gender discordance, and duplicates. | Reference method at Uppsala University Hospital |
| SHIP-Trend | Prospective population-based study | 986 | none | Excluded arrays with CallRate < 94%, duplicate samples (by estimated IBD) and individuals with reported/genotyped gender mismatch | Blood samples in SHIP-Trend were taken, while subjects were on random salt diet and under regular medication, from fasting subjects in supine position. Serum calcium has been measured by photometric rocedure of bichromatic endpoint measurement on an Dimension Vista system (Dade Behring, Eschborn, Germany). The analytical measurement range was: 1.25-3.75 mmol/L. During the course of the study the inter-assay coefficient of variation was < 5%. |
| TwinsUK | Twin Study | 3965 | none | Of the 5,654 participants who underwent genotyping, we made the following exclusions: >3 standard deviations and missing informations. This resulted in a total of 3965 genotyped individuals. | Assays for serum calcium was performed using standard laboratory procedures. The test was performed on a 950 Vitros analyser (Ortho-Clinical Diagnostics; Johnson and Johnson, Rochester, NY, U.S.A.). |
| The BRItish Genetics of HyperTension (BRIGHT) study | Hypertensive cases from the BRIGHT study resource. | 2000 | BMI>35  diabetes, secondary hypertension or a co-existing illness. | Of 2000 cases typed, we excluded individuals if they had >3% missing data or evidence of non-European ancestry under eigenstrat analysis, n=277. | Serum calcium measures were performed on non-fasting samples by the Clinical Biochemistry Unit at the University of Glasgow |

**Additional file 2**. *P* Values for Associations of 8 Calcium-Associated Genetic Variants with 8 known PD risk factors

| SNP | Pos (hg19) | Trait | Study | PMID | P | N |
| --- | --- | --- | --- | --- | --- | --- |
| rs780094 | chr2:27741237 | BMI | GIANT | 25673413 | 2.05E-04 | 322044 |
| rs7481584 | chr11:3029089 | BMI | GIANT | 25673413 | 0.02849 | 232503 |
| rs17711722 | chr7:65271197 | BMI | GIANT | 25673413 | 0.0455 | 233688 |
| rs10491003 | chr10:9328651 | BMI | GIANT | 25673413 | 0.221 | 222260 |
| rs7336933 | chr13:42559076 | BMI | GIANT | 25673413 | 0.5586 | 233962 |
| rs1801725 | chr3:122003757 | BMI | GIANT | 25673413 | 0.5841 | 322043 |
| rs1550532 | chr2:234264848 | BMI | GIANT | 25673413 | 0.6527 | 233743 |
| rs1570669 | chr20:52774427 | BMI | GIANT | 25673413 | 0.7077 | 233663 |
| rs7336933 | chr13:42559076 | Body fat percentage | Lu Y | 26833246 | 0.149 | 76060 |
| rs780094 | chr2:27741237 | Body fat percentage | Lu Y | 26833246 | 0.3475 | 100639 |
| rs10491003 | chr10:9328651 | Body fat percentage | Lu Y | 26833246 | 0.4367 | 70397 |
| rs1570669 | chr20:52774427 | Body fat percentage | Lu Y | 26833246 | 0.4751 | 75815 |
| rs1801725 | chr3:122003757 | Body fat percentage | Lu Y | 26833246 | 0.5163 | 100653 |
| rs17711722 | chr7:65271197 | Body fat percentage | Lu Y | 26833246 | 0.6364 | 74256 |
| rs1550532 | chr2:234264848 | Body fat percentage | Lu Y | 26833246 | 0.654 | 76121 |
| rs7481584 | chr11:3029089 | Body fat percentage | Lu Y | 26833246 | 0.7346 | 76086 |
| rs1801725 | chr3:122003757 | Cigarettes per day | TAG | 20418890 | 0.09868 | 38181 |
| rs780094 | chr2:27741237 | Cigarettes per day | TAG | 20418890 | 0.2184 | 38181 |
| rs17711722 | chr7:65271197 | Cigarettes per day | TAG | 20418890 | 0.2361 | 38181 |
| rs1550532 | chr2:234264848 | Cigarettes per day | TAG | 20418890 | 0.4318 | 38181 |
| rs7336933 | chr13:42559076 | Cigarettes per day | TAG | 20418890 | 0.457 | 38181 |
| rs1570669 | chr20:52774427 | Cigarettes per day | TAG | 20418890 | 0.487 | 38181 |
| rs7481584 | chr11:3029089 | Cigarettes per day | TAG | 20418890 | 0.5377 | 38181 |
| rs10491003 | chr10:9328651 | Cigarettes per day | TAG | 20418890 | 0.9987 | 38181 |
| rs780094 | chr2:27741237 | Continuous (log10 grams/day) alcohol traits | AlcGen and CHARGE + | 27911795 | 3.65E-09 | 70460 |
| rs1550532 | chr2:234264848 | Continuous (log10 grams/day) alcohol traits | AlcGen and CHARGE + | 27911795 | 0.386 | 70460 |
| rs7481584 | chr11:3029089 | Continuous (log10 grams/day) alcohol traits | AlcGen and CHARGE + | 27911795 | 0.393 | 70460 |
| rs1570669 | chr20:52774427 | Continuous (log10 grams/day) alcohol traits | AlcGen and CHARGE + | 27911795 | 0.4071 | 70460 |
| rs10491003 | chr10:9328651 | Continuous (log10 grams/day) alcohol traits | AlcGen and CHARGE + | 27911795 | 0.6499 | 70460 |
| rs17711722 | chr7:65271197 | Continuous (log10 grams/day) alcohol traits | AlcGen and CHARGE + | 27911795 | 0.6723 | 70460 |
| rs1801725 | chr3:122003757 | Continuous (log10 grams/day) alcohol traits | AlcGen and CHARGE + | 27911795 | 0.7526 | 70460 |
| rs7336933 | chr13:42559076 | Continuous (log10 grams/day) alcohol traits | AlcGen and CHARGE + | 27911795 | 0.8629 | 70460 |
| rs7336933 | chr13:42559076 | DBP | ICBP | 21909115 | 0.0553 | 69395 |
| rs1550532 | chr2:234264848 | DBP | ICBP | 21909115 | 0.118 | 69395 |
| rs7481584 | chr11:3029089 | DBP | ICBP | 21909115 | 0.397 | 69395 |
| rs780094 | chr2:27741237 | DBP | ICBP | 21909115 | 0.544 | 69395 |
| rs17711722 | chr7:65271197 | DBP | ICBP | 21909115 | 0.563 | 69395 |
| rs10491003 | chr10:9328651 | DBP | ICBP | 21909115 | 0.921 | 69395 |
| rs1801725 | chr3:122003757 | DBP | ICBP | 21909115 | 0.969 | 69395 |
| rs1570669 | chr20:52774427 | DBP | ICBP | 21909115 | 0.982 | 69395 |
| rs1801725 | chr3:122003757 | Ever smoker | TAG | 20418890 | 6.62E-03 | 74035 |
| rs10491003 | chr10:9328651 | Ever smoker | TAG | 20418890 | 0.03622 | 74035 |
| rs780094 | chr2:27741237 | Ever smoker | TAG | 20418890 | 0.07861 | 74035 |
| rs17711722 | chr7:65271197 | Ever smoker | TAG | 20418890 | 0.2317 | 74035 |
| rs7481584 | chr11:3029089 | Ever smoker | TAG | 20418890 | 0.433 | 74035 |
| rs1550532 | chr2:234264848 | Ever smoker | TAG | 20418890 | 0.6549 | 74035 |
| rs7336933 | chr13:42559076 | Ever smoker | TAG | 20418890 | 0.6687 | 74035 |
| rs1570669 | chr20:52774427 | Ever smoker | TAG | 20418890 | 0.6844 | 74035 |
| rs1801725 | chr3:122003757 | Former smoker | TAG | 20418890 | 0.03506 | 41278 |
| rs1570669 | chr20:52774427 | Former smoker | TAG | 20418890 | 0.1388 | 41278 |
| rs1550532 | chr2:234264848 | Former smoker | TAG | 20418890 | 0.4342 | 41278 |
| rs17711722 | chr7:65271197 | Former smoker | TAG | 20418890 | 0.4365 | 41278 |
| rs10491003 | chr10:9328651 | Former smoker | TAG | 20418890 | 0.5326 | 41278 |
| rs780094 | chr2:27741237 | Former smoker | TAG | 20418890 | 0.5729 | 41278 |
| rs7336933 | chr13:42559076 | Former smoker | TAG | 20418890 | 0.8203 | 41278 |
| rs7481584 | chr11:3029089 | Former smoker | TAG | 20418890 | 0.8593 | 41278 |
| rs780094 | chr2:27741237 | HDL | GLGC | 24097068 | 2.67E-03 | 187006 |
| rs7481584 | chr11:3029089 | HDL | GLGC | 24097068 | 7.73E-03 | 94311 |
| rs1801725 | chr3:122003757 | HDL | GLGC | 24097068 | 0.08756 | 187094 |
| rs1550532 | chr2:234264848 | HDL | GLGC | 24097068 | 0.5915 | 94302 |
| rs17711722 | chr7:65271197 | HDL | GLGC | 24097068 | 0.6227 | 92696 |
| rs7336933 | chr13:42559076 | HDL | GLGC | 24097068 | 0.6748 | 85432 |
| rs10491003 | chr10:9328651 | HDL | GLGC | 24097068 | 0.8139 | 94311 |
| rs1570669 | chr20:52774427 | HDL | GLGC | 24097068 | 0.8393 | 92771 |
| rs780094 | chr2:27741237 | Hip circumference | GIANT | 25673412 | 3.40E-05 | 212961 |
| rs17711722 | chr7:65271197 | Hip circumference | GIANT | 25673412 | 0.091 | 145325 |
| rs7481584 | chr11:3029089 | Hip circumference | GIANT | 25673412 | 0.11 | 144031 |
| rs1801725 | chr3:122003757 | Hip circumference | GIANT | 25673412 | 0.29 | 212962 |
| rs1570669 | chr20:52774427 | Hip circumference | GIANT | 25673412 | 0.5 | 145326 |
| rs10491003 | chr10:9328651 | Hip circumference | GIANT | 25673412 | 0.69 | 133713 |
| rs7336933 | chr13:42559076 | Hip circumference | GIANT | 25673412 | 0.81 | 145425 |
| rs1550532 | chr2:234264848 | Hip circumference | GIANT | 25673412 | 0.95 | 145345 |
| rs780094 | chr2:27741237 | LDL | GLGC | 24097068 | 1.02E-07 | 172941 |
| rs10491003 | chr10:9328651 | LDL | GLGC | 24097068 | 0.04724 | 89888 |
| rs1801725 | chr3:122003757 | LDL | GLGC | 24097068 | 0.2526 | 173022 |
| rs7481584 | chr11:3029089 | LDL | GLGC | 24097068 | 0.4794 | 89888 |
| rs7336933 | chr13:42559076 | LDL | GLGC | 24097068 | 0.5353 | 81123 |
| rs1570669 | chr20:52774427 | LDL | GLGC | 24097068 | 0.6041 | 88384 |
| rs1550532 | chr2:234264848 | LDL | GLGC | 24097068 | 0.772 | 89879 |
| rs17711722 | chr7:65271197 | LDL | GLGC | 24097068 | 0.9417 | 87149 |
| rs1550532 | chr2:234264848 | SBP | ICBP | 21909115 | 0.113 | 69395 |
| rs1801725 | chr3:122003757 | SBP | ICBP | 21909115 | 0.261 | 69395 |
| rs7336933 | chr13:42559076 | SBP | ICBP | 21909115 | 0.269 | 69395 |
| rs780094 | chr2:27741237 | SBP | ICBP | 21909115 | 0.313 | 69395 |
| rs7481584 | chr11:3029089 | SBP | ICBP | 21909115 | 0.684 | 69395 |
| rs10491003 | chr10:9328651 | SBP | ICBP | 21909115 | 0.956 | 69395 |
| rs1570669 | chr20:52774427 | SBP | ICBP | 21909115 | 0.962 | 69395 |
| rs17711722 | chr7:65271197 | SBP | ICBP | 21909115 | 0.986 | 69395 |
| rs780094 | chr2:27741237 | Serum urate | GUGC | 23263486 | 6.52E-39 | 110160 |
| rs7481584 | chr11:3029089 | Serum urate | GUGC | 23263486 | 0.0332 | 109350 |
| rs1550532 | chr2:234264848 | Serum urate | GUGC | 23263486 | 0.09339 | 109948 |
| rs17711722 | chr7:65271197 | Serum urate | GUGC | 23263486 | 0.2013 | 109847 |
| rs1801725 | chr3:122003757 | Serum urate | GUGC | 23263486 | 0.2207 | 110201 |
| rs10491003 | chr10:9328651 | Serum urate | GUGC | 23263486 | 0.627 | 109902 |
| rs1570669 | chr20:52774427 | Serum urate | GUGC | 23263486 | 0.6882 | 109729 |
| rs7336933 | chr13:42559076 | Serum urate | GUGC | 23263486 | 0.9504 | 102909 |
| rs780094 | chr2:27741237 | Total cholesterol | GLGC | 24097068 | 5.28E-41 | 187196 |
| rs7481584 | chr11:3029089 | Total cholesterol | GLGC | 24097068 | 0.1224 | 94595 |
| rs10491003 | chr10:9328651 | Total cholesterol | GLGC | 24097068 | 0.1747 | 94595 |
| rs7336933 | chr13:42559076 | Total cholesterol | GLGC | 24097068 | 0.2404 | 85718 |
| rs1801725 | chr3:122003757 | Total cholesterol | GLGC | 24097068 | 0.2504 | 187289 |
| rs1550532 | chr2:234264848 | Total cholesterol | GLGC | 24097068 | 0.3782 | 94586 |
| rs17711722 | chr7:65271197 | Total cholesterol | GLGC | 24097068 | 0.5407 | 91847 |
| rs1570669 | chr20:52774427 | Total cholesterol | GLGC | 24097068 | 0.7294 | 93018 |
| rs780094 | chr2:27741237 | Triglycerides | GLGC | 20686565 | 7.08E-125 | 96598 |
| rs17711722 | chr7:65271197 | Triglycerides | GLGC | 20686565 | 0.01201 | 96598 |
| rs1550532 | chr2:234264848 | Triglycerides | GLGC | 20686565 | 0.2927 | 96598 |
| rs7481584 | chr11:3029089 | Triglycerides | GLGC | 20686565 | 0.4333 | 96598 |
| rs7336933 | chr13:42559076 | Triglycerides | GLGC | 20686565 | 0.5006 | 96598 |
| rs1570669 | chr20:52774427 | Triglycerides | GLGC | 20686565 | 0.5168 | 96598 |
| rs10491003 | chr10:9328651 | Triglycerides | GLGC | 20686565 | 0.5753 | 96598 |
| rs1801725 | chr3:122003757 | Triglycerides | GLGC | 20686565 | 0.5783 | 96598 |
| rs780094 | chr2:27741237 | Type II diabetes | DIAGRAM | 24509480 | 1.00E-05 | 110452 |
| rs7481584 | chr11:3029089 | Type II diabetes | DIAGRAM | 24509480 | 0.015 | 110452 |
| rs1570669 | chr20:52774427 | Type II diabetes | DIAGRAM | 24509480 | 0.097 | 110452 |
| rs1801725 | chr3:122003757 | Type II diabetes | DIAGRAM | 24509480 | 0.35 | 110452 |
| rs17711722 | chr7:65271197 | Type II diabetes | DIAGRAM | 24509480 | 0.46 | 110452 |
| rs1550532 | chr2:234264848 | Type II diabetes | DIAGRAM | 24509480 | 0.58 | 110452 |
| rs10491003 | chr10:9328651 | Type II diabetes | DIAGRAM | 24509480 | 0.83 | 110452 |
| rs7336933 | chr13:42559076 | Type II diabetes | DIAGRAM | 24509480 | 0.87 | 110452 |
| rs1570669 | chr20:52774427 | Vitamin D | Jiang | 29343764 | 5.33E-06 | 79366 |
| rs10491003 | chr10:9328651 | Vitamin D | Jiang | 29343764 | 0.1094 | 79366 |
| rs1801725 | chr3:122003757 | Vitamin D | Jiang | 29343764 | 0.1282 | 79366 |
| rs17711722 | chr7:65271197 | Vitamin D | Jiang | 29343764 | 0.159 | 79366 |
| rs1550532 | chr2:234264848 | Vitamin D | Jiang | 29343764 | 0.3112 | 79366 |
| rs7481584 | chr11:3029089 | Vitamin D | Jiang | 29343764 | 0.5405 | 79366 |
| rs780094 | chr2:27741237 | Vitamin D | Jiang | 29343764 | 0.8382 | 79366 |
| rs7336933 | chr13:42559076 | Vitamin D | Jiang | 29343764 | 0.9071 | 79366 |
| rs780094 | chr2:27741237 | Waist circumference | GIANT | 25673412 | 0.015 | 232032 |
| rs7481584 | chr11:3029089 | Waist circumference | GIANT | 25673412 | 0.064 | 152532 |
| rs17711722 | chr7:65271197 | Waist circumference | GIANT | 25673412 | 0.13 | 153817 |
| rs10491003 | chr10:9328651 | Waist circumference | GIANT | 25673412 | 0.17 | 142210 |
| rs1570669 | chr20:52774427 | Waist circumference | GIANT | 25673412 | 0.41 | 153696 |
| rs1801725 | chr3:122003757 | Waist circumference | GIANT | 25673412 | 0.51 | 232014 |
| rs1550532 | chr2:234264848 | Waist circumference | GIANT | 25673412 | 0.59 | 153840 |
| rs7336933 | chr13:42559076 | Waist circumference | GIANT | 25673412 | 0.91 | 153917 |
| rs10491003 | chr10:9328651 | Waist hip ratio | GIANT | 25673412 | 0.041 | 132859 |
| rs7481584 | chr11:3029089 | Waist hip ratio | GIANT | 25673412 | 0.15 | 143173 |
| rs7336933 | chr13:42559076 | Waist hip ratio | GIANT | 25673412 | 0.4 | 144565 |
| rs780094 | chr2:27741237 | Waist hip ratio | GIANT | 25673412 | 0.48 | 212136 |
| rs17711722 | chr7:65271197 | Waist hip ratio | GIANT | 25673412 | 0.49 | 144465 |
| rs1801725 | chr3:122003757 | Waist hip ratio | GIANT | 25673412 | 0.51 | 212143 |
| rs1550532 | chr2:234264848 | Waist hip ratio | GIANT | 25673412 | 0.96 | 144488 |
| rs1570669 | chr20:52774427 | Waist hip ratio | GIANT | 25673412 | 0.98 | 144490 |
| rs780094 | chr2:27741237 | Waist hip ratio adjusted for BMI | GIANT | 25673412 | 1.80E-03 | 224328 |
| rs10491003 | chr10:9328651 | Waist hip ratio adjusted for BMI | GIANT | 25673412 | 0.044 | 130256 |
| rs7481584 | chr11:3029089 | Waist hip ratio adjusted for BMI | GIANT | 25673412 | 0.24 | 139770 |
| rs1801725 | chr3:122003757 | Waist hip ratio adjusted for BMI | GIANT | 25673412 | 0.26 | 224337 |
| rs17711722 | chr7:65271197 | Waist hip ratio adjusted for BMI | GIANT | 25673412 | 0.63 | 142386 |
| rs1570669 | chr20:52774427 | Waist hip ratio adjusted for BMI | GIANT | 25673412 | 0.67 | 142595 |
| rs7336933 | chr13:42559076 | Waist hip ratio adjusted for BMI | GIANT | 25673412 | 0.78 | 142703 |
| rs1550532 | chr2:234264848 | Waist hip ratio adjusted for BMI | GIANT | 25673412 | 0.91 | 142658 |

The significance threshold for the association of these 8 variants with these known and potential confounders is *P* < 0.00625 (a Bonferroni correction, *P* < 0.05/8). Highlighted values are *P* < 0.00625.

**Additional file 3**. Individual causal estimates from each of serum calcium genetic variants and PD using different methods in Discovery PD GWAS dataset.


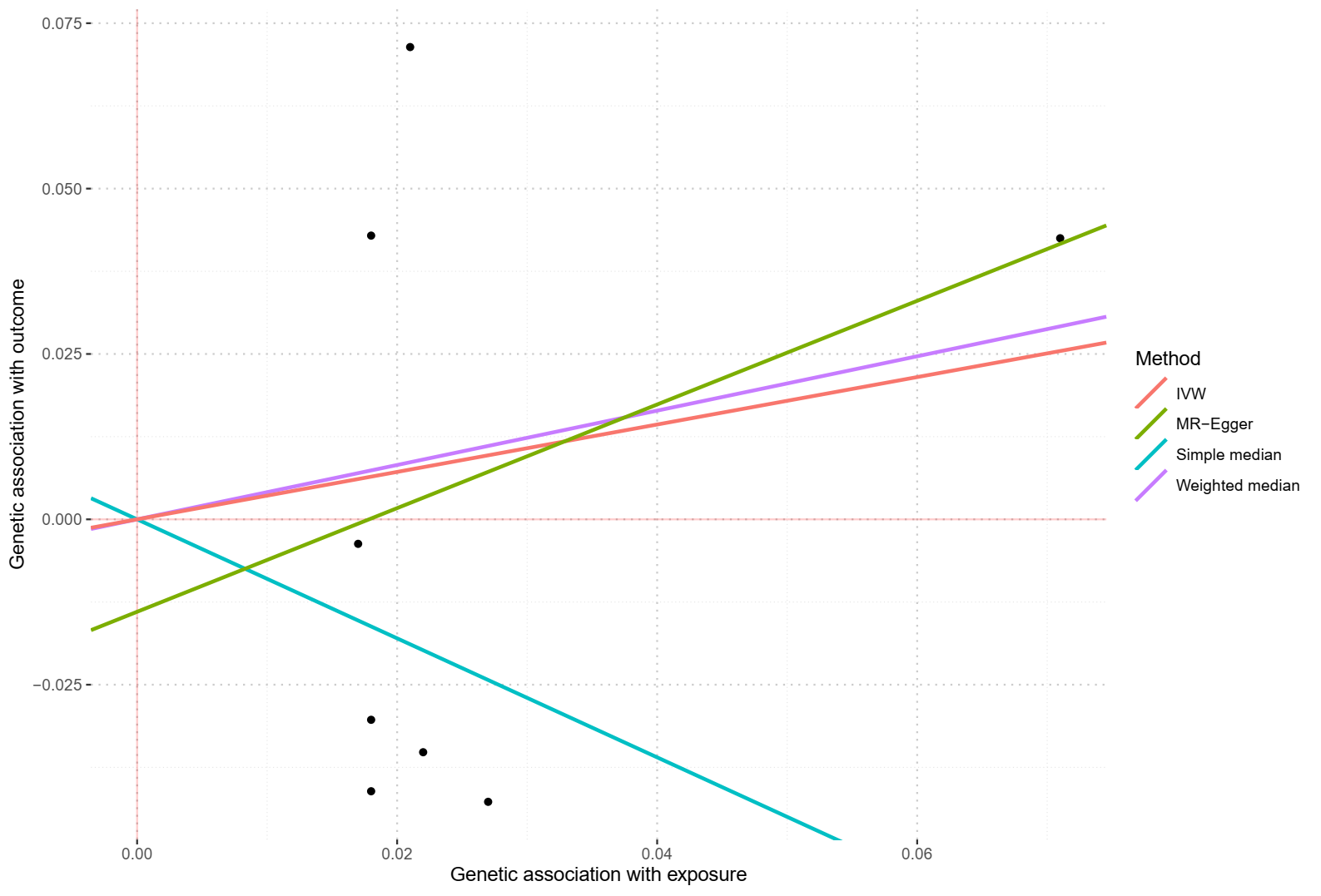


**Additional file 4**. Individual causal estimates from each of serum calcium genetic variants and PD using different methods in Validation PD GWAS dataset.


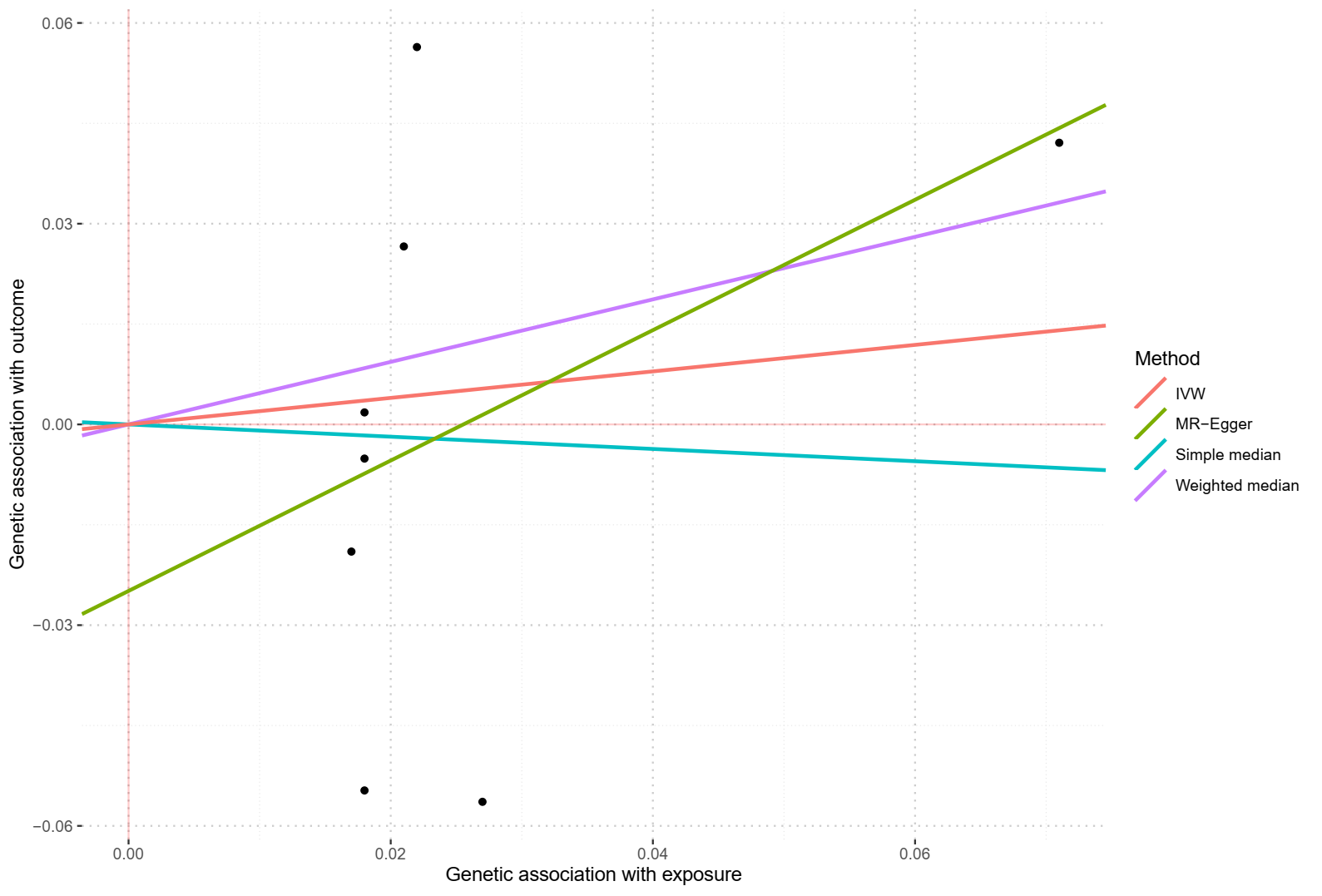


**Additional file 5**. Forest plot for MR estimates about the association between genetically increased serum calcium levels and PD in Discovery PD GWAS dataset.


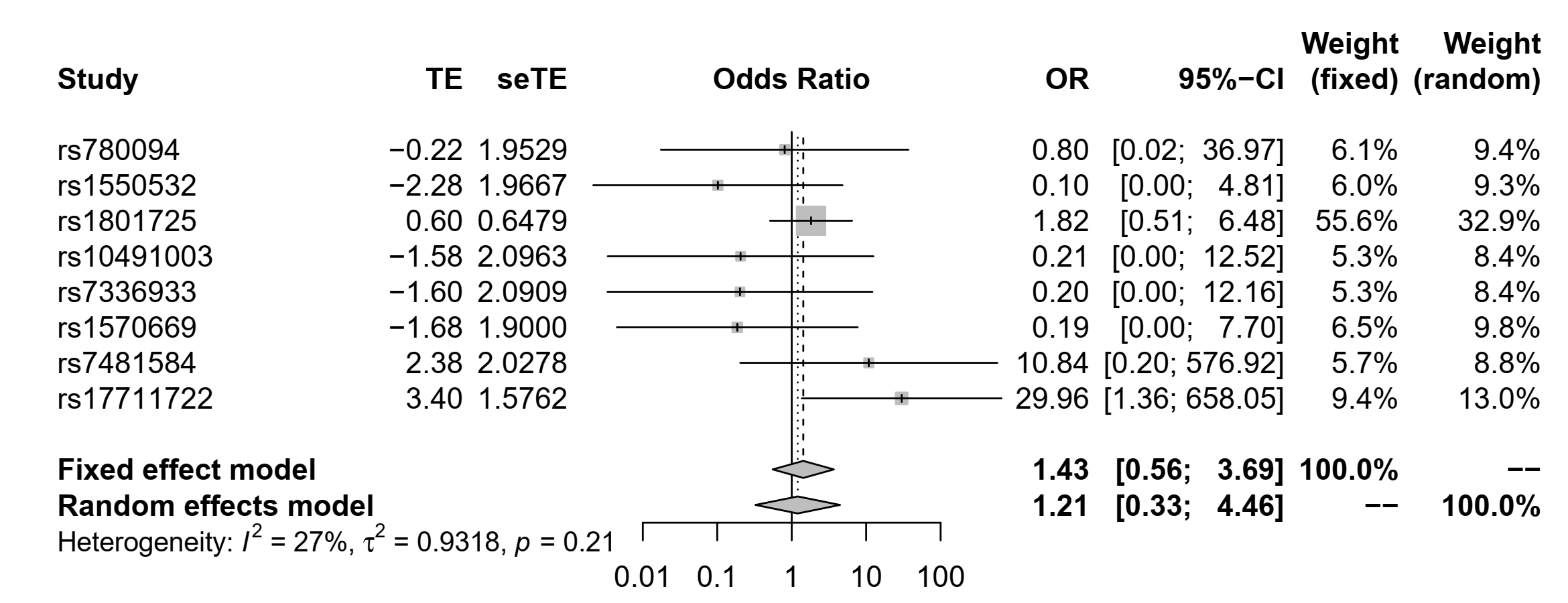


**Additional file 6**. Forest plot for MR estimates about the association between genetically increased serum calcium levels and PD in Validation PD GWAS dataset.


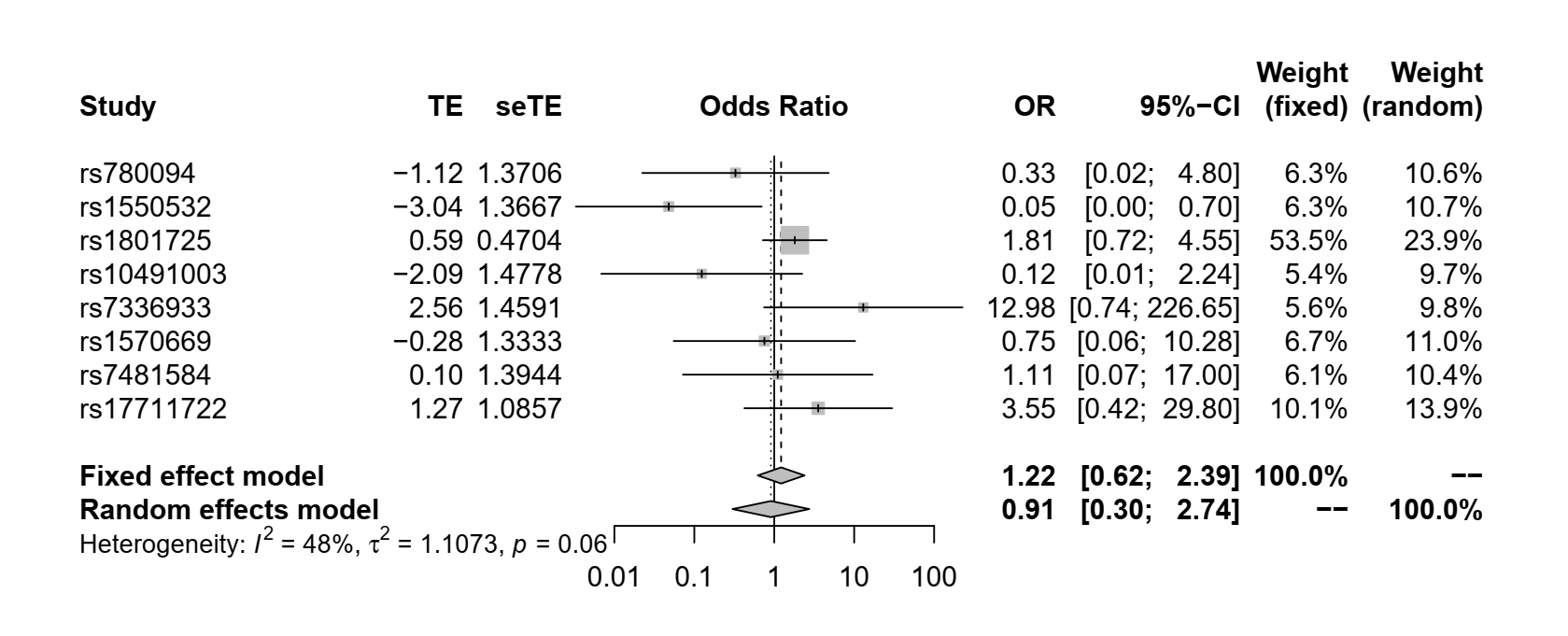


**Reference**

1. O'Seaghdha CM, Wu H, Yang Q, Kapur K, Guessous I, Zuber AM, et al. Meta-analysis of genome-wide association studies identifies six new Loci for serum calcium concentrations. PLoS Genet. 2013;9(9):e1003796.
